# Biocompatibility and interaction of porous alumina-zirconia scaffolds with adipose-derived mesenchymal stem cells for bone tissue regeneration

**DOI:** 10.1016/j.heliyon.2023.e20128

**Published:** 2023-09-13

**Authors:** Atanasio S. Vidane, Fabio C. Nunes, Julieta A. Ferreira, Heidge Fukumasu, Silvio H. Freitas, Eliria MJA. Pallone, Carlos E. Ambrósio

**Affiliations:** a Department of Clinics, Veterinary Faculty, Eduardo Mondlane University, Maputo, Mozambique; b Department of Biosystems Engineering, Faculty of Animal Science and Food Engineering, University of São Paulo, São Paulo, Brazil; c Department of Veterinary Medicine, Faculty of Animal Science and Food Engineering, University of São Paulo, São Paulo, Brazil

**Keywords:** Alumina-zirconia, Biomaterial, Cell therapy, Osteointegration, Porous ceramic

## Abstract

Replacement of bone defects with bone graft or implant is an important therapeutic strategy that has been used in routine practice. However, the identification of biomaterials that can mimic natural bone properties and serve as bone substitutes remains a major challenge. In this context, alumina-zirconia (Al_2_O_3_/ZrO_2_) nanocomposites emerge as potential alternatives for biomedical applications, owing to their high mechanical strength, wear resistance, and biocompatibility. In this sense, in this study, we prepared porous Al_2_O_3_/ZrO_2_ nanocomposites (scaffolds) using the gelcasting method and biomimetically coated them with calcium phosphate (CaP). We evaluated the biocompatibility of the scaffolds using the quantitative MTT cytotoxicity test in L929 cells. Moreover, rabbit adipose-derived mesenchymal stem cells (rADMSCs) were seeded with CaP-containing and CaP-free porous samples to evaluate cell proliferation and cell–scaffold interaction *in vitro*. Our results showed that the Al_2_O_3_/ZrO_2_ scaffolds were non-cytotoxic, and there were no significant differences between CaP-containing and CaP-free scaffolds in terms of cell growth and adhesion. In contrast, when co-cultured with rADMSCs, the scaffolds enhanced cell proliferation and cell adhesion. The rADMSCs adhered and migrated through the pores of the scaffold and anchored to different poles with differentiated elongated structures. These results suggest osteogenic differentiation of rADMSCs in response to mechanical loading of Al_2_O_3_/ZrO_2_ scaffolds. Therefore, we conclude that Al_2_O_3_/ZrO_2_ scaffolds have demonstrated significant implications in bone tissue engineering and are valuable biomaterials for bone replacement.

## Highlights

• Al_2_O_3_/ZrO_2_ scaffolds demonstrated high biocompatibility and non-cytotoxicity *in vitro*.
• Comparable cell growth and adhesion observed in CaP-containing and CaP-free scaffolds.
• Scaffolds enhanced cell proliferation, adhesion, and osteogenic differentiation of rADMSCs.
• Al_2_O_3_/ZrO_2_ scaffolds show promise for bone tissue engineering applications.

## Graphical abstract

**Figure:**
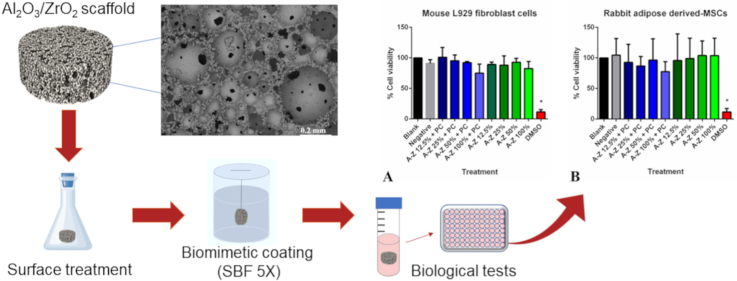

## 1 Introduction

Orthopedic disorders related to bone loss caused by clinical situations such as fractures, bone neoplasms, congenital defect, or non-union fractures are of critical concern in both veterinary and human medicine [ [[1], [2], [3]]]. Replacement of bone defects with bone graft or implant is an important therapeutic strategy that has been used in routine practice. Autologous bone graft is considered the best standard clinical material for bone regeneration in terms of osteoconduction and osteoinduction. However, its clinical practice is limited due to its low availability and long surgical procedures [ [4,5]]. Using autologous or heterologous bone grafts can lead to damage, often caused by infections, additional surgeries, or extended, painful post-operative healing. This highlights the urgent requirement for developing synthetic biomaterials with superior regenerative potential [6].

Orthopedic tissue engineering or biomaterials science is a very active multidisciplinary research area, which is involved in addressing the problem of bone loss and creating new possibilities for repairing skeletal tissues using synthetic bone substitutes [ [1,4,[7], [8], [9]]]. However, the identification of biomaterials that can mimic natural bone properties and serve as bone substitutes remains a major challenge. In general, synthetic biomaterials should be biocompatible, biodegradable, nonallergenic, nonimmunogenic, nonmutagenic, and porous, and must promote differentiation of stem cells into bone cell lineages [ [1,2,10]].

Among synthetic grafts, bioceramics are known to have suitable mechanical properties that mimic the natural properties of bone [ [2,5,10]]. Al_2_O_3_/ZrO_2_ composites are bio-inert ceramics that have high biocompatibility, flexural strength, and toughness. This attractive set of properties makes the Al_2_O_3_/ZrO_2_ composites appealing for biomedical applications, where the composites can serve as substrates. These materials can be combined with bioactive materials using coatings [ [11,12]]. On the other hand, calcium phosphate (CaP) is a bioactive ceramic that plays a key role in several biological processes including bone tissue regeneration [ [4,13,14]]. CaPs are the main inorganic elements in bones. They are widely used in regenerative therapies due to their biocompatibility and osteoconductive properties, facilitating new bone tissue formation. Meanwhile, CaPs can also be absorbed slowly by the body over time, gradually replacing damaged tissue [ [4,14,15]].

In order to obtain a high porous biomaterial, the gelcasting method was used. Gelcasting combines a gas phase with a ceramic mixture containing ceramic powder, water, deflocculants, binders, and gelling agents. The material is then solidified through in situ copolymerization of organic monomers after foam formation, followed by drying and sintering. The resulting porous network has sturdy walls, spherical pores, and appealing mechanical properties [16].

Tissue regeneration also involves intense cellular interactions and activities. The mesenchymal stem cells (MSCs) play a key role in this process due to their high capability to proliferate and differentiate into specialized lineages in response to specific stimuli. The adipose tissue is recognized as an important source of MSCs; these adipose-derived MSCs (ADMSCs) exhibit high potential for treatment of several medical conditions [17]. Though the osteogenic potential of ADMSCs *in vitro* and *in vivo* has been discussed previously [ [[18], [19], [20], [21]]], the mechanism by which ADMSCs exert their therapeutic effects is not well understood. Researchers have attributed these effects to: (i) self-renewal ability of ADMSCs; (ii) ability of ADMSCs to differentiate into specialized cell lineages that can directly mediate tissue repair; (iii) transport of mRNA, microRNA, protein, and membrane receptors by microvesicles/exosomes, thereby mediating cell-to-cell communication; and (iv) activation and mediation of regulatory progenitor cells, immune cells, blood cells, and endothelial cells [ [17,22,23]].

The aim of this study was to produce Al_2_O_3_/ZrO_2_ scaffolds coated with CaP and evaluate their biocompatibility and interaction with rabbit ADMSCs (rADMSCs) *in vitro*. Thus, the cell viability, adhesion, and proliferation were evaluated. Combining the properties of Al_2_O_3_ and ZrO_2_ with that of CaP will aid in producing a porous optimal biomaterial similar to natural bone and provide satisfactory mechanical conditions to drive cell adhesion, proliferation, and differentiation. We believe that rADMSCs will improve implant microenvironment, enhance osteoconductive properties, and promote osteoinduction and osteogenicity.

## 2 Materials and methods

This study was approved by the Faculty of Animal Science and Food Engineering (ethical committee protocol number: CEUA 5442150419; ID 001195). All animal experiments were carried out in accordance with the U.K. Animals (Scientific Procedures) Act, 1986 and associated guidelines, EU Directive 2010/63/EU for animal experiments, or the National Institutes of Health guide for the care and use of Laboratory animals (NIH Publications No. 8023, revised 1978).

### 2.1 Preparation of Al_2_O_3_/ZrO_2_ scaffolds

For preparation of nanocomposites, commercial Al_2_O_3_ powder (AKP-53, 99.995% purity) with 0.2 μm particle size (Sumitomo Chemical Co., Japan) and nanometric ZrO_2_ powder (99.9% purity), partially stabilized with 3% yttria and having an average particle size of 50 nm (Nanostructured Materials Inc), were used. Al_2_O_3_ suspension was prepared by ball milling using 0.2 wt% *para*-aminobenzoic acid (PABA) in alcohol medium for 1 h, with ball-to-powder mass ratio of 5:1. Nanometric ZrO_2_ suspension was prepared by ball milling using 0.5 wt% PABA in alcohol medium for 12 h, with ball-to-powder mass ratio of 4:1. The mixed suspension that comprised of 5 vol% of previously prepared ZrO_2_ in Al_2_O_3_ suspension was milled for 22 h. Next, 0.5 wt% of oleic acid was added to the final suspension and milled for further 2 h. The resulting mixture was air-dried at 25 °C and calcined at an increasing heating rate (1 °C per min to 400 °C) for 1 h [24].

The Al_2_O_3_/ZrO_2_ scaffolds were prepared by combining gelcasting and foaming techniques under a non-controlled atmosphere [25]. A ceramic suspension of 40 vol% Al_2_O_3_/ZrO_2_ powders and 30 vol% organic hydroxymethylacrylamide, methacrylamide, and methylenobisacrylamide (3:3:1 M ratio), was dispersed in ammonium polymethacrylate and deagglomerated using the conventional ball milling method, with ball-to-powder mass ratio of 3:1. A non-ionic foaming agent (Lutensol FSA 10), polymerization initiator (ammonium persulfate), and catalyst (N,N,N'N'-tetramethylethylenediamine) were added to the resulting mixture with constant stirring [16].

The samples were molded into circular shapes (diameter-to-height ratio of 2:1), calcined at 600 °C for 2 h, sintered at 1500 °C for 2 h [ [11,16,26]], and cut into small cubes of dimension 0.4 × 0.4 × 0.3 mm.

The Al_2_O_3_/ZrO_2_ scaffolds were treated with 5 M H_3_PO_4_ solution in a thermostatic bath and incubated at 90 °C for 4 days [ [11,15,25]]. Next, the scaffolds were biomimetically coated with CaP [12], according to the procedure described by Barrere [12], wherein the ionic content of simulated body fluid (SBF) was 5 times greater than that of original. The concentration of ions in SBF 5 × was 733.5 nM NaCl, 7.5 nM MgCl_2_·6H_2_O, 12.5 nM CaCl_2_·2H_2_O, 5.0 nM Na_2_HPO_4_·2H_2_O, and 21.0 nM NaHCO_3_, pH = 6.10. After 14 days of incubation at 36.5 °C, the scaffolds were removed from SBF, washed with ultra pure water, and dried at 50 °C for 24 h. After the incubation time in the biomimetic coating, a layer of CaP was formed onto the surface of the scaffolds.

The apparent porosity (%) of the scaffolds was determined using mercury intrusion porosimetry. The penetrometer (AutoPore IV 9500, Micromeritics, USA) was evacuated at a pressure of 7.10–6 MPa (∼50 μmHg), followed by mercury filling at a pressure of 413.68 MPa. The scaffolds (before and after biomimetic coating) were structurally characterized using scanning electron microscopy (SEM; HITACHI TM 3000 M). The CaP layer formed on the scaffolds was evaluated using X-ray diffraction (XRD; RIGAKU MINIFLEX 600) at a scan range of 31.0°–34.5° and scan step of 0.20°. The diffractograms were normalized and mathematically treated for baseline correction, and curve deconvolution analysis was performed using the Gaussian function (R^2^ > 0.99) of the Origin 9.0.0 software (OriginLab).

### 2.2 Cytotoxicity assays

The biocompatibility of Al_2_O_3_/ZrO_2_ scaffolds was evaluated using MTT assay (3-dimethylthiazol-2,5-diphenyl tetrazolium bromide), an indirect standard colorimetric method, as described previously [27]. Briefly, cryopreserved mouse fibroblast L929 (ATTC CCL-1) cells were thawed and cultured until confluence in Dulbecco's modified Eagle medium/F-12 Ham's (DMEM/F12; Gibco) supplemented with 10% fetal bovine serum (FBS; Gibco), and 1% penicillin and streptomycin solution (P/S; Invitrogen). The cells were recovered using 0.25% trypsin (TrypLE express, Gibco) and counted using the trypan blue dye method (1:1).

Simultaneously, the CaP-containing and CaP-free scaffolds were surgically packed, autoclaved, and incubated with the previously described culture medium at a concentration of 0.1 g/mL for 24 h at 37 °C under 5% CO_2_ atmosphere [28]. Standard DMEM/F12 without biomaterial was used as control.

Passage 3 cells were seeded in 96-well tissue culture plates at a density of 3 × 10^3^ cells/well and incubated at 37 °C and 5% CO_2_ (eighteen replicates). After 24 h of incubation, the culture medium in each well was replaced with different dilutions (1, ½, ¼, ⅛) of the scaffolds extracts and incubated at 37 °C and 5% CO_2_ for 24 h. Dimethyl sulfoxide (DMSO; 25%, Sigma Aldrich) was used as positive control (reproducible cytotoxic response), and aluminum oxide was used as negative control. Next, 50 μL of MTT solution (Sigma Aldrich) was added to each well to obtain a final concentration of 5 mg/mL and incubated at 37 °C and 5% CO_2_ for 2 h. The formazan crystals were dissolved by adding 100 μL of isopropanol (Synth, L 202805). Absorbance was measured using a microplate reader (Fluostar Optima BMD, Labtech) at 540 ± 10 nm. Cell viability was calculated using the formula: viability (%) = (ae540/ab540)*100, where ae represents the mean absorbance of sample and ab represents the mean absorbance of blank.

### 2.3 Isolation of rADMSCs

ADMSCs were isolated from abdominal subcutaneous adipose tissue, excised from three adult male New Zealand rabbits. Pieces of adipose tissue were mechanically minced and subjected to enzymatic digestion with 0.1% collagenase (Gibco) at 38.5 °C. The cell suspension was centrifuged at 303 g for 5 min at 25 °C. The supernatant was discarded and the cells were cultured until confluence in Alpha minimum essential medium (MEM, Gibco) supplemented with 10% FBS, 1% P/S, 1% l-glutamine, 1% non-essential amino acids, and 1% amphotericin at 38.5 °C and 5% CO_2_. Next, the cells were recovered using 0.25% trypsin and counted using the trypan blue dye method (1:1). The rADMSCs were frozen below passage two in MEM supplemented with 45% FBS and 10% DMSO. The cells were frozen for 24 h at −80 °C, followed by storage in liquid nitrogen. All rADMSCs used in this study were of passage 3 or 4.

### 2.4 Viability of rADMSCs in Al_2_O_3_/ZrO_2_ scaffolds

The viability of rADMSCs in Al_2_O_3_/ZrO_2_ scaffolds was evaluated using MTT assay as previously described, with the following modifications: (i) cryopreserved cells were thawed and cultured until confluence in Alpha MEM supplemented with 10% FBS, 1% P/S, 1% l-glutamine, 1% non-essential amino acids, and 1% amphotericin; (ii) standard MEM without biomaterial was used as control.

### 2.5 Adhesion and proliferation of rADMSCs

The rADMSCs were seeded with CaP-containing or CaP-free Al_2_O_3_/ZrO_2_ scaffolds in 24-well culture plates (10^6^ cells/well) containing Alpha MEM supplemented with 10% FBS, 1% P/S, 1% l-glutamine, 1% non-essential amino acids, and 1% amphotericin. The cells were incubated at 38.5 °C and 5% of CO_2_, and the medium was replaced every 48 h over a period of 28 days (the duration of the study). Cell morphology and proliferation were examined every day using an inverted phase contrast microscope (TCM400 Labomed).

To investigate cell–substrate interaction, the rADMSCs were fixed in 2.5% glutaraldehyde for 24 h, followed by dehydration in graded concentrations of ethanol (40, 60, 80, and 100%). The samples were dried in a desiccator at 25 °C room temperature and observed using SEM.

### 2.6 Statistical analyses

All experiments were performed in triplicate for all tested parameters. All results are expressed as the arithmetic mean ± standard deviation. Groups were compared using one-way analysis of variance (One-way ANOVA). Differences between the groups were analyzed using Tukey's test. Levels of *p* < 0.05 was considered statistically significant. All statistical analyses were performed using GraphPad 6 statistical software program.

## 3 Results

### 3.1 Microstructure of the Al_2_O_3_/ZrO_2_ scaffolds

**Fig. 1:**
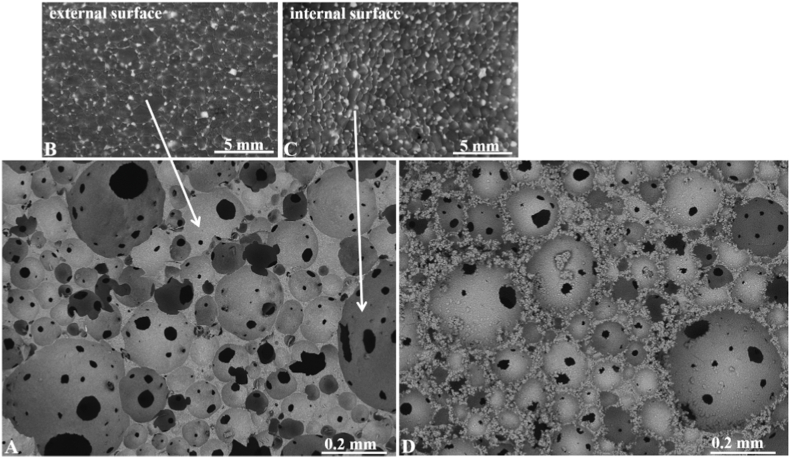
SEM images of the surface of Al_2_O_3_/ZrO_2_ scaffolds. (A) Overview of the polished surface; (B) magnified outer surface of a pore; (C) magnified inner surface of a pore; (D) SEM image of the Al_2_O_3_/ZrO_2_ scaffold after 14 days of biomimetic coating.

SEM images of the surfaces of Al_2_O_3_/ZrO_2_ scaffolds prior to biomimetic coating in SBF 5 × solution are illustrated in Fig. 1 (A–C). Fig. 1A shows a highly porous structure with good spatial distribution of spherical pores. The darkest spots observed in Fig. 1A indicate the presence of intra-pores, suggesting high interconnectivity.

Fig. 1B and C shows the SEM images of the outer and inner pore surfaces, respectively. It was observed that ZrO_2_ inclusions (lighter regions) were evenly distributed in the Al_2_O_3_ matrix. In addition, the ZrO_2_ inclusions were found in the grain boundaries including triple points, which favor the pinning effect.

Fig. 1D shows the SEM image of the Al_2_O_3_/ZrO_2_ porous sample after biomimetic coating in SBF 5 × solution for 14 days. In general, globular crystal apatite clusters were observed on the outer and inner pore surfaces.

**Fig. 2:**
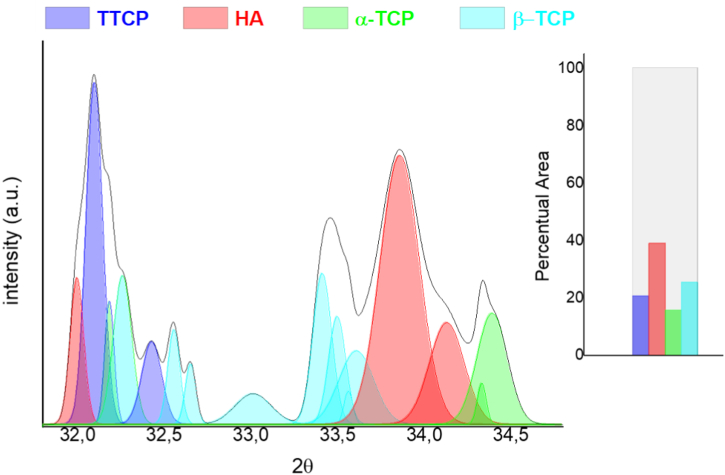
XRD patterns of calcium phosphates formed on the surface of chemically treated porous Al_2_O_3_/ZrO_2_ scaffold after 14 days of biomimetic coating. The graph shows the percentage of the total area for each calcium phosphate phase present, obtained from curve deconvolution analysis. The phases formed were: α-tricalcium phosphate (α-TCP), β-tricalcium phosphate (β-TCP), hydroxyapatite (HA), and tetracalcium phosphate (TTCP).

Fig. 2 illustrates the XRD pattern of CaP formed on the scaffold surface. The CaP phases were identified and quantified using curve deconvolution analysis (Table 1). The curve displacements were characterized based on standard Miller indices (*hkl*) for each CaP phase, according to the Joint Committee for Powder Diffraction Studies (JCPDS) files 09–432, 73–1731, 09–348, 09–169, and 25–1137.

**Table 1:** Area (%) of calcium phosphate phases formed on the surface of Al_2_O_3_/ZrO_2_ scaffolds after 14 days of incubation.

Calcium phosphates	Miller Indices^a^	Area (%)
TTCP	(113) (023) (−212)	20.47
HA	(211) (300) (202)	38.81
α-TCP	(530) (043)	15.57
β-TCP	(128) (306) (1112)	25.15

a Standard Miller Indices (*hkl*) in accordance with the database provided by the Joint Committee on Powder Diffraction Standards, International Center for Diffraction Data, and American Society for Testing and Materials, Powder Diffraction File (2017), card numbers: 09–432, 73–1731, 09–348, 09–169, 25–1137.

In general, four different phases of CaP were observed on the scaffold surface: α-tricalcium phosphate (α-TCP) planes (530)/(043), β-tricalcium phosphate (β-TCP) planes (128)/(306)/(1112), hydroxyapatite (HA) planes (211)/(300)/(202), and tetracalcium phosphate (TTCP) planes (113)/(023)/(−212). The major phase was HA phase (38.81%), followed by β-TCP (25.15%), TTCP (20.47%), and α-TCP (15.57%) phases.

### 3.2 Cytotoxicity of Al_2_O_3_/ZrO_2_ scaffolds

**Fig. 3:**
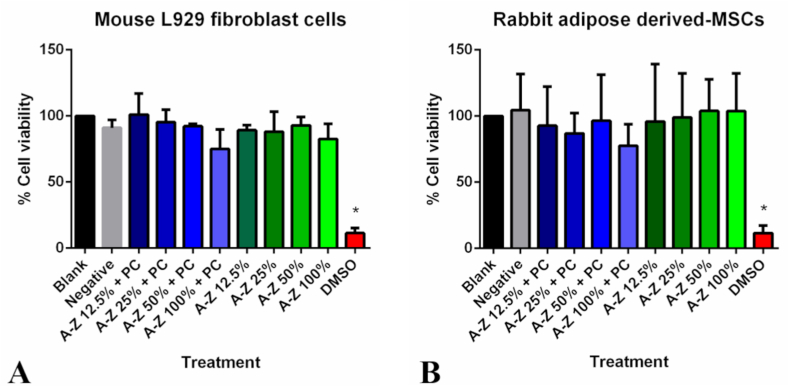
**Determination of cytotoxicity of Al**_**2**_**O**_**3**_**/ZrO**_**2**_**scaffolds using MTT assay.** The CaP-containing and CaP-free Al_2_O_3_/ZrO_2_ scaffolds did not affect cell viability or proliferation of mouse L929 cells (A) and rabbit adipose-derived MSCs (B).

**Fig. 4:**
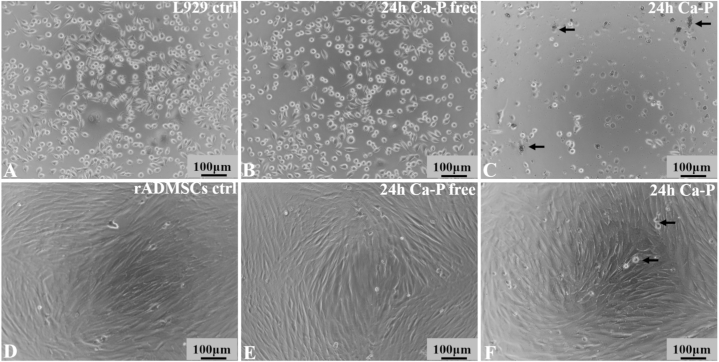
**Photomicrographs of mouse L929 cells (A**–**C) and rabbit adipose-derived MSCs (D**–**F).** The cell morphology did not change 24 h after culturing with CaP-containing and CaP-free Al_2_O_3_/ZrO_2_ scaffolds as compared to the control. In CaP-containing scaffolds (C and F), presence of soluble CaP crystals (black arrows) was observed.

The biocompatibility of Al_2_O_3_/ZrO_2_ scaffold was quantitatively estimated using MTT assay in L-929 cells. The results of MTT assay showed that addition of 100% of CaP-containing or CaP-free Al_2_O_3_/ZrO_2_ scaffold did not affect cell viability or proliferation (Fig. 3A). No changes in cell morphology were observed during the entire duration of the experiment (Fig. 4A–C). However, cell viability was lower in CaP-containing scaffold as compared to CaP-free, but the difference was not significant. Blank control was considered as 100% cell viability.

### 3.3 Viability of rADMSCs in Al_2_O_3_/ZrO_2_ scaffolds

The rADMSCs adhered to the culture plate after 24 h of culture in Alpha MEM and reached about 80% confluence in 48 h. The cells showed a fibroblast-like morphology, indistinguishable from bone marrow-derived MSCs (Fig. 4D). After third passage, the isolated cells reached sufficient level of homogeneity. The cryopreservation process did not alter the cell morphology or proliferative ability.

Cell viability in Al_2_O_3_/ZrO_2_ scaffold was quantitatively measured using MTT assay. Addition of 100% of CaP-containing or CaP-free Al_2_O_3_/ZrO_2_ scaffold did not alter cell viability or proliferation (Fig.s. 3B & 4D–F). Flow cytometric analysis revealed that rADMSCs expressed MSC-specific surface markers (CD90, CD73, and CD100) and were negative for hematopoietic markers (CD34 and CD45). Under specific culture conditions, the cells underwent osteogenic, chondrogenic, and adipogenic differentiation (Data not shown, previously published) [29].

### 3.4 Adhesion and proliferation of rADMSCs

**Fig. 5:**
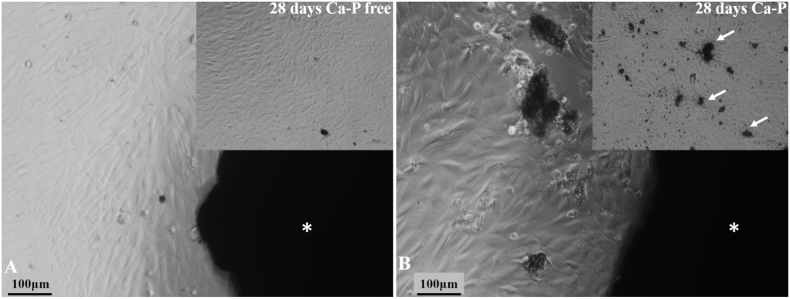
**Photomicrographs of the interaction of rADMSCs with CaP-free (A) and CaP-containing (B) porous Al**_**2**_**O**_**3**_**/ZrO**_**2**_**scaffolds.** The scaffolds (*) did not affect the surrounding cell morphology or proliferation rate after 24 h. The cells grew towards the scaffold and clumped together around it. In CaP-containing scaffolds (B), presence of soluble CaP crystals (white arrows) was observed.

The rADMSCs were seeded with CaP-containing and CaP-free Al_2_O_3_/ZrO_2_ scaffold to evaluate cell morphology, cell proliferation, and cell–substrate interaction *in vitro*. We observed that the scaffold did not alter the cell morphology or proliferative ability at all tested time-points (Fig. 5A and B). In addition, clumping of rADMSCs around the scaffold was observed.

**Fig. 6:**
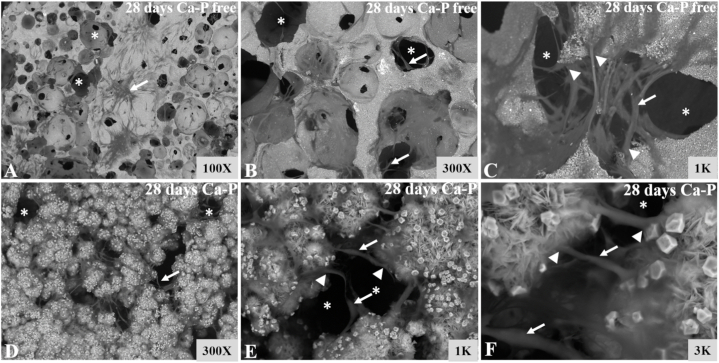
**SEM images of CaP-free (A**–**C) and CaP-containing (D**–**F) porous Al**_**2**_**O**_**3**_**/ZrO**_**2**_**scaffolds.** The cells penetrated and migrated through the tunnels formed by Al_2_O_3_/ZrO_2_ scaffold pores (*). The cells (white arrows) presented a fusiform morphology with several cytoplasmic processes (white head arrows) that interconnected with other cells as well as the biomaterial surface.

SEM images showed that the cells adhered well to the surface of Al_2_O_3_/ZrO_2_ scaffolds. Furthermore, the images revealed that the cells penetrated and migrated through the tunnels formed by biomaterial pores. The cells presented a fusiform morphology with several cytoplasmic processes that interconnected with other cells as well as the scaffold surface (Fig. 6 A–F). The cell morphology and cell–scaffold interaction pattern were not affected by CaP coating.

## 4 Discussion

The Al_2_O_3_/ZrO_2_ scaffolds have a recognized potential as bone graft substitutes owing to the high hardness of Al_2_O_3_ and excellent toughness of ZrO_2_. CaP coating improves the bioactivity as well as the biointegration and biodegradation properties of the scaffold. In this study, we produced a highly porous Al_2_O_3_/ZrO_2_ scaffold coated with CaP. The scaffold showed good spatial distribution of pores with high interconnectivity, which is the key factor in designing bone substitutes. Scaffolds mimic the bone tissue, and the pores allow vascular invasion, effective nutrient diffusion, and metabolic flow during the bone healing and regeneration process [ [9,16]]. Pore size is a key factor for cell viability and affinity, as pores influence cell migration, communication, intracellular signaling, and flow of nutrients and metabolites [ [[30], [31], [32]]]. The scaffolds produced in this study had 56.38 ± 0.34% porosity, which is favorable for normal biological events in the bone [33].

Although the chemical surface modification was performed, improving the interface between the scaffold and CaPs deposited by the biomimetic method is still a challenge. Moreover, the samples produced by the gelcasting method can exhibit less mechanical resistance when compared with other techniques, such as additive manufacturing and sol-gel [34].

SEM images showed that ZrO_2_ inclusions were evenly distributed in the Al_2_O_3_ matrix. In addition, the ZrO_2_ inclusions were also found in grain boundaries including triplex points, which favor the pinning effect. This pinning effect inhibits Al_2_O_3_ matrix grain growth [24].

The XRD pattern revealed different phases of CaP on the scaffold surface. The hydroxyl groups (OH^−^) on the scaffold surface provide favorable sites for nucleation of apatite, thereby promoting apatite formation [ [35,36]]. Preliminary surface treatment using biomimetic coating method can stimulate the formation of functional OH^−^ groups on scaffold surface, significantly promoting the growth of apatite when scaffolds are immersed in SBF. Presence of nanometric ZrO_2_ stimulates apatite nucleation owing to the interactions between Zr–OH and apatite clusters [ [11,26]].

Firstly, we investigated the *in vitro* biocompatibility of the Al_2_O_3_/ZrO_2_ scaffolds. Cell viability was quantitatively evaluated using an indirect standard colorimetric method, MTT assay, according to International Standard Organization [27]. The results showed that the Al_2_O_3_/ZrO_2_ scaffolds were non-cytotoxic to both L929 cells and rADMSCs. Therefore, these scaffolds are compatible with biological systems and can be safely used as bone substitutes. However, in both L929 cells and rADMSCs, CaP-containing scaffolds tend to reduce cell viability as compared to CaP-free nanoparticles (not significant). In solution, the CaP particles detached from the scaffolds and caused slight physical damage to the cells. Previous studies have also reported that the adhesion of CaP coating using the biomimetic method is poor [37] and is strongly affected by the ionic strength of the solution [ [11,12]]. Al_2_O_3_/ZrO_2_ scaffolds are widely considered the best option for bone grafts because they closely mimic the bone tissue and are biocompatible [ [13,25]]. CaP is an essential compound found in living organisms, and it participates in several key biological processes. Therefore, CaP coating is fundamental to obtain improved bioactivity properties and to enhance osteoconduction and osteoinduction, which are essential for the tissue regeneration process [ [15,38,39]].

Secondly, we investigated the growth behavior and interaction of CaP-containing and CaP-free Al_2_O_3_/ZrO_2_ scaffolds with rADMSCs. Instead of growing the cells on the surface of the biomaterial, we placed the scaffolds on the edge of the culture plate. We observed a strong interaction between the scaffolds and the rADMSCs. The cells were strongly attracted and adhered to the scaffolds, and they migrated through the pores of the biomaterial. Interestingly, the cells that came in contact with the biomaterial completely changed their morphology. The cells presented a fusiform morphology with several cytoplasmic processes that interconnected with other cells as well as the biomaterial surface. Based on this observation, we speculate that the cells have begun the osteogenic differentiation process in response to the typical bone microenvironment provided by the Al_2_O_3_/ZrO_2_ scaffolds; this phenomenon will be further investigated thoroughly by our team in future. The differentiation of MSCs into osteogenic cell lineages in response to biomaterial load has been reported in several recent studies and is generally attributed to the interactions between cells and the biomaterial surface [ [[40], [41], [42], [43], [44], [45], [46], [47], [48], [49]]]. In addition, several other molecules, such as integrins and bone morphogenetic proteins (BMPs), are also involved in the osteogenic differentiation of MSCs [ [[50], [51], [52], [53]]], but the underlying mechanism is not yet understood clearly.

## 5 Conclusions

In this study, we demonstrated that CaP-containing and CaP-free Al_2_O_3_/ZrO_2_ scaffolds are non-cytotoxic and highly biocompatible with rADMSCs. Our results suggest that enrichment of Al_2_O_3_/ZrO_2_ scaffolds with MSCs can improve osteoconduction, osteoinduction, and osteointegration. However, the influence of scaffolds on the osteogenic differentiation of MSCs must be thoroughly investigated. The combination of high biocompatibility, improved osteogenic properties and advantageous mechanical properties makes this biomaterial an attractive option for the development of novel bone regeneration therapies.

## Author contribution statement

Atanasio Vidane, Fabio C Nunes, Julieta A Ferreira: Conceived and designed the experiments; Performed the experiments; Analyzed and interpreted the data; Wrote the paper.

Heidge Fukumasu: Performed the experiments; Analyzed and interpreted the data; Contributed reagents, materials, analysis tools or data.

Silvio H Freitas: Conceived and designed the experiments; Performed the experiments; Analyzed and interpreted the data; Contributed reagents, materials, analysis tools or data; Wrote the paper.

Eliria M.J.A. Pallone, Carlos Eduardo Ambrósio: Conceived and designed the experiments; Analyzed and interpreted the data; Contributed reagents, materials, analysis tools or data; Wrote the paper.

## Funding statement

Atanasio Vidane was supported by 10.13039/501100003593Conselho Nacional de Desenvolvimento Científico e Tecnológico {22/05031-0}.

Professor Carlos Eduardo Ambrósio was supported by 10.13039/501100001807Fundação de Amparo à Pesquisa do Estado de São Paulo {17/21266-0}.

Atanasio Vidane was supported by 10.13039/501100002222The World Academy of Sciences {167026/2018-6}.

Fabio C Nunes was supported by 10.13039/501100002322Coordenação de Aperfeiçoamento de Pessoal de Nível Superior {001}.

Professor Carlos Eduardo Ambrósio was supported by 10.13039/501100001807Fundação de Amparo à Pesquisa do Estado de São Paulo {2022/10640-0}.

## Data availability statement

Data will be made available on request.

## Declaration of competing interest

The authors declare that they have no known competing financial interests or personal relationships that could have appeared to influence the work reported in this paper.
